# Who and when should we screen for prostate cancer? Interviews with key opinion leaders

**DOI:** 10.1186/s12916-015-0526-x

**Published:** 2015-11-27

**Authors:** Sigrid Carlsson, Michael Leapman, Peter Carroll, Fritz Schröder, Peter C. Albertsen, Dragan Ilic, Michael Barry, Dominick L. Frosch, Andrew Vickers

**Affiliations:** Department of Surgery (Urology Service), Memorial Sloan Kettering Cancer Center, 485 Lexington Avenue, New York, 10017 NY USA; Department of Urology, Institute of Clinical Sciences, Sahlgrenska Academy at University of Göteborg, Göteborg, Sweden; Department of Urology, University of California, San Francisco, CA USA; Department of Urology, Erasmus University Medical Center, Rotterdam, The Netherlands; University of Connecticut Health Center, 263 Farmington Avenue, Farmington, 06030 CT USA; Department of Epidemiology & Preventive Medicine, Monash University, Level 6, The Alfred Centre, 99 Commercial Rd, Melbourne, VIC 3004 Australia; The Informed Medical Decisions Foundation, Boston, MA USA; Gordon and Betty Moore Foundation, Palo Alto, CA & Department of Medicine, University of California, Los Angeles, USA; Department of Epidemiology & Biostatistics, Memorial Sloan Kettering Cancer Center, 485 Lexington Avenue, New York, 10017 NY USA

## Abstract

Prostate cancer screening using prostate-specific antigen (PSA) is highly controversial. In this Q & A, Guest Editors for *BMC Medicine*’s ‘Spotlight on Prostate Cancer’ article collection, Sigrid Carlsson and Andrew Vickers, invite some of the world’s key opinion leaders to discuss who, and when, to screen for prostate cancer. In response to the points of view from the invited experts, the Guest Editors summarize the experts’ views and give their own personal opinions on PSA screening.

## Introduction

**Fig. 1:**
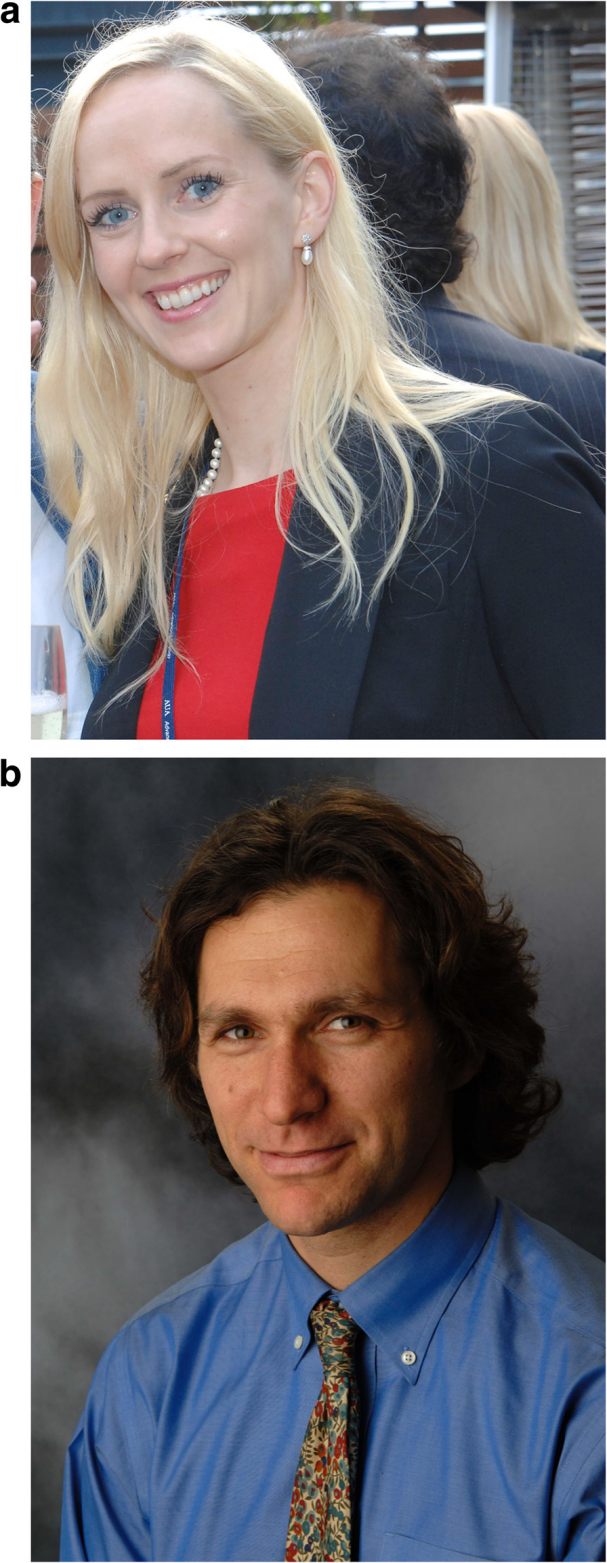
Sigrid Carlsson and Andrew Vickers are the guest editors of *BMC Medicine*’s ‘Spotlight on Prostate Cancer’ article collection. Sigrid Carlsson (**a**) is an associate professor of experimental urology with a PhD in medicine (urology); she has an MPH from Harvard School of Public Health. Andrew Vickers (**b**) holds a DPhil in clinical medicine and specializes in research methodology and statistics. Both editors have more than 10 years of experience in the study of screening, early detection, and treatment of prostate cancer and are on staff at Memorial Sloan Kettering Cancer Center, New York, NY, USA

**Sigrid Carlsson (Fig.****1a****) and Andrew Vickers (Fig.****1b****)**

Screening for prostate cancer with prostate-specific antigen (PSA) is controversial. Screening is currently transitioning from being an all-or-nothing-question, to finding new ways of individualized testing. However, consensus remains to be reached within guideline groups and worldwide experts regarding who – and when – to screen, if at all. In this Q & A, we invite seven of the world’s key opinion leaders in the field, both proponents and skeptics, to elaborate on what they believe the current screening policy should be. The authors have all published widely on PSA, and comprise a wide variety of experience in areas such as urology, epidemiology, evidence-based medicine, and medical decision-making.

Currently, only one guideline group, the United States Preventive Services Task Force (USPSTF), recommends against screening for all men [1]. Most other guideline groups recommend shared decision-making, involving a discussion of the pros and cons of screening [2]. To aid in decision-making, some propose using a risk-stratified approach taking into account multiple factors along with a PSA measurement [3]. However, the specifics of such an approach are a subject of debate; for instance, the appropriate age limits of screening remain to be defined. Randomized screening trials, including the European Randomized Study of Screening for Prostate Cancer (ERSPC) and the Göteborg trial [4, 5] have provided evidence that regular PSA-screening can reduce prostate cancer mortality by 21–44 % at 13–14 years of follow-up; the age groups studied in these trials were 55–69 and 50–64 years, respectively. Thus, the question remains regarding the screening of men outside this age range. There is a growing body of evidence on the benefits of commencing screening in the mid-40s. While the American Urological Association (AUA) bases its recommendation on the 55–69 age group based on the ERSPC results [6], the European Urological Association recommends a baseline PSA be obtained at 40–45 years of age [7].

Our personal view is that PSA screening should indeed involve shared decision-making, but we believe the focus should primarily be on behavior, rather than preference. For this purpose, we have published a decision-support tool called the ‘Simple Schema’ [8], which acknowledges that the majority of harms of screening result from unnecessary treatment of low-risk disease and therefore focuses on the importance of active surveillance as the appropriate, evidence-based management strategy for low-risk cancer [9–11]. We further believe that PSA screening should be a risk-stratified approach aimed at detecting lethal prostate cancer. This is based on evidence that only a small proportion of men with moderately elevated PSA have aggressive disease [5] and that overdiagnosis is strongly influenced by age and PSA levels. For instance, we have shown that almost half of the excess incidence of cancer associated with PSA testing occurs in men over 70 [12] – a group in which screening is likely of little, if any, benefit [5, 13] – and that the effects of screening men in their 60s is highly dependent on their PSA level, with an excellent ratio of harms to benefits in patients with PSA ≥2 ng/mL but zero benefit in patients with a lower PSA [14]. Therefore, the current guidelines in place at Memorial Sloan Kettering Cancer Center restrict screening in men over 60 to those with above average PSAs and dramatically restrict screening in men over 70 to a small number of men with exceptional health and high PSA [15]. Additionally, biopsy is recommended only after a repeat PSA and further work-up, and the frequency of screening is stratified depending on baseline PSA, which has been shown to be a very strong predictor of long-term prostate cancer metastasis and death [16–18].

We were interested in hearing the experts elaborate on these nuances of screening as we believe this would help us forward in striving to identify the group of men who might benefit from screening and those who might not.

## Who and when to screen, and not to screen, for prostate cancer: the proponents’ view

We asked these authors, generally perceived as proponents of prostate cancer screening, what they think current policy for prostate cancer screening should be. We then asked some follow-up questions.

**Fig. 2:**
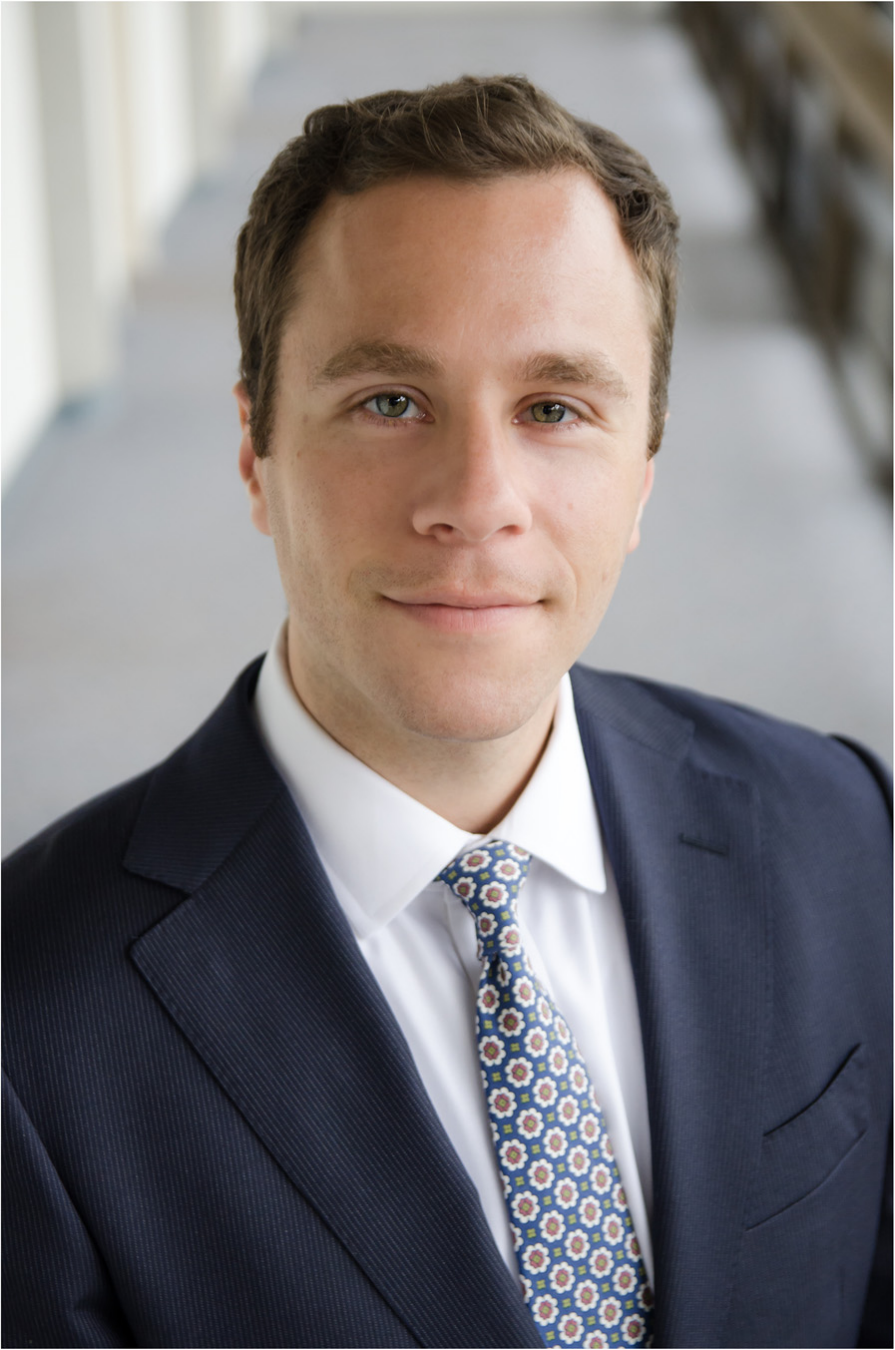
Michael Leapman, MD, is currently a urologic oncology fellow at the University of California, San Francisco. He completed his urology residency at the Mount Sinai Hospital in New York. His research interests are in developing novel risk prediction tools in early stage prostate cancer

**Fig. 3:**
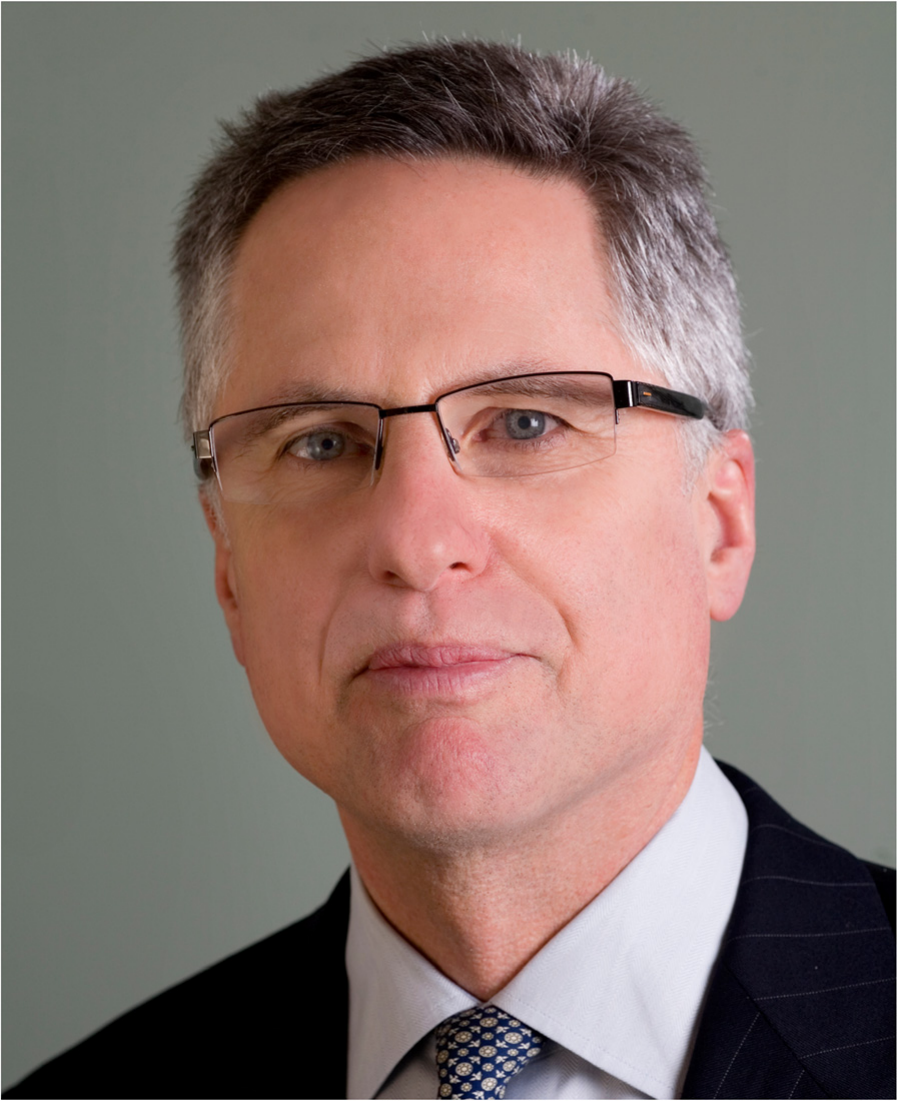
Peter Carroll, MD, MPH, is the Ken and Donna Derr-Chevron Distinguished Professor and Chair of the Department of Urology at the University of California, San Francisco. Dr. Carroll is a recognized leader in the field of prostate cancer and urologic oncology, and has authored or co-authored over 500 publications. His main research interests include identifying clinical and pathologic determinants of prostate cancer recurrence, progression, and mortality, discovering novel biomarkers for prostate cancer diagnostics and prognostics, developing evidence-based guidelines for improved management of prostate cancer patients, and examining the impact of lifestyle on health-related quality of life and survivorship among men with prostate cancer

**Fig. 4:**
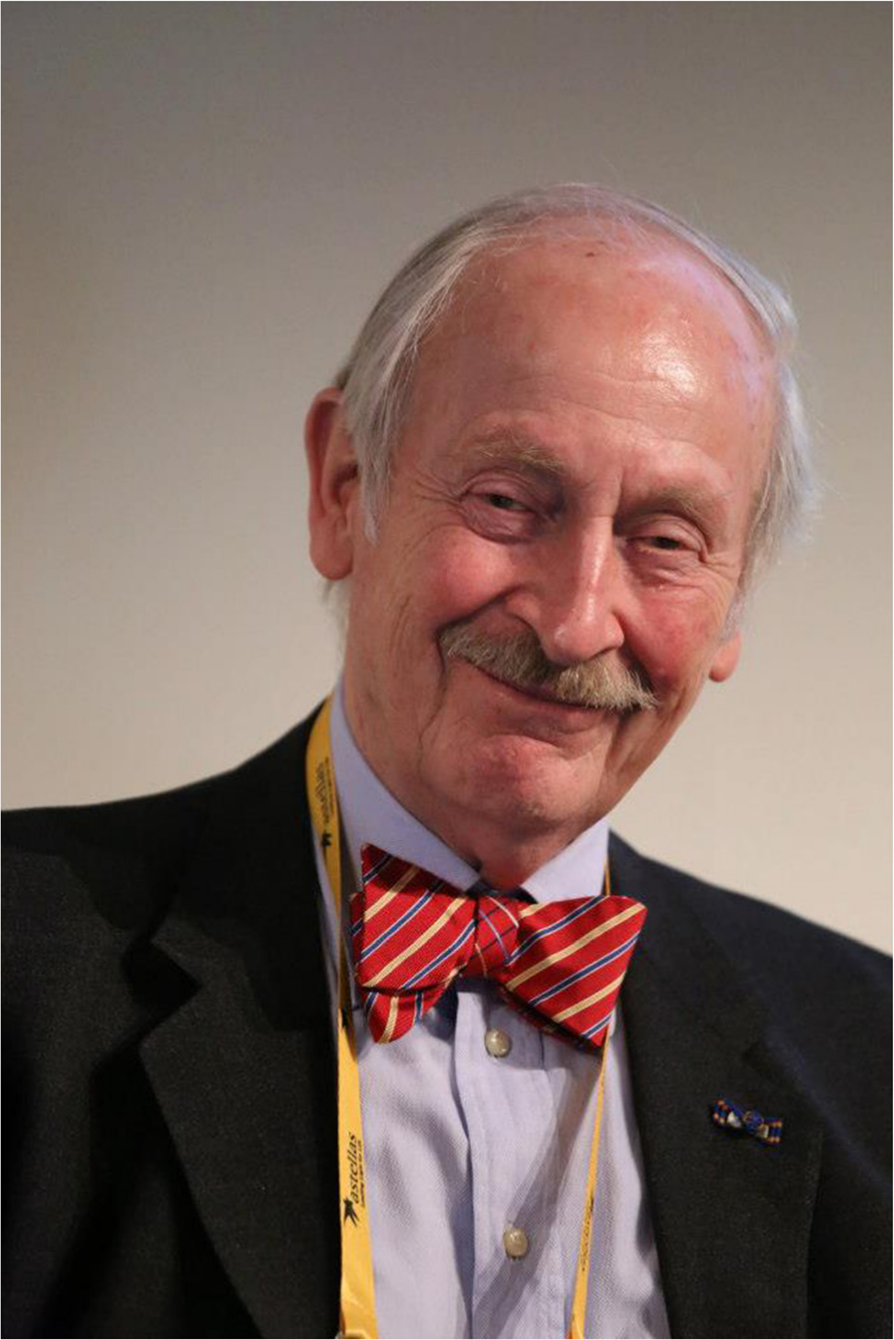
Fritz Schröder is Professor of Urology at the Erasmus Medical Center, the Netherlands*.* He was international coordinator of the ERSPC and led the development of the Société Internationale D’Urologie (SIU) decision aid for PSA testing

**Michael Leapman (Fig.** **2****), Peter Carroll (Fig.** **3****), and Fritz Schröder (Fig.** **4****)**

**A Vickers and S Carlsson: You have generally been perceived as proponents of prostate cancer screening. What do you think current policy should be; should all men be screened regardless of symptoms, age, and baseline PSA levels? Justify your answer with reference to the literature.**

**M Leapman & P Carroll:** Prostate cancer is a highly prevalent disease that exhibits considerable variation in clinical behavior; some men will enjoy long lives with low-grade disease without treatment, while a significant, but smaller number may succumb to a devastating metastatic burden. Therefore, the current challenge faced in the early management of prostate cancer is to identify and treat disease in men likely to obtain benefit while sparing unnecessary detection and, most importantly, treatment in those who will not.

Compelling randomized evidence suggests that PSA screening of asymptomatic men is associated with a significant reduction in death from prostate cancer. The recent 13-year update of the ERSPC trial, a multi-centered study examining prostate cancer mortality among participants aged 50–74 receiving regular PSA screening compared with no routine screening, has demonstrated a relative risk of death from prostate cancer of 0.79 (95 % CI, 0.69–0.91, *P* = 0.001) in favor of screening men aged 55–69 years. Moreover, when adjusting for non-participation, the reduction in risk increased to 27 % (95 % CI, 0.61–0.88, *P* < 0.0007) [5]. Naturally, screening can only reduce mortality by effecting treatment of clinically significant disease, as was observed in the ERSPC experience, in which substantially more cancers were diagnosed and subsequently treated [19].

It is true that eliminating PSA screening will obviously decrease the number of ‘insignificant’ prostate cancer cases detected; however, this would come at the imprudent expense of ignoring disease in intermediate and high-risk patients who may stand to benefit substantially. Indeed, the rationale for treatment in appropriate patients is redoubled by randomized evidence suggesting improvement in survival and metastatic progression with timely treatment of threatening cancers [20]. However, an overall improvement in prostate cancer mortality is alone insufficient to justify expansive screening and treatment of all men if such a strategy will expose those harboring non-lethal tumors to a non-trivial risk of adverse quality of life outcomes; one should not screen if overdiagnosis is followed by overtreatment [21].

Ultimately, the landscape of early prostate cancer detection may not truly be cast in a monochromatic decision palette, namely to screen with PSA or not to screen. Rational practices including the screening of healthy men with a long life expectancy starting at age 45, cessation of screening in those with significant co-morbidity and those of advanced age, extending the interval of screening in most men (2–4 years), and discontinuing screening for those with low-risk profiles at certain ages alone would significantly improve the efficiency of early detection. In addition, nuanced strategies for prostate cancer detection and management represent an auspicious frontier. Although not validated in randomized trials of screening techniques, assays incorporating novel PSA isoforms – including the 4-Kallikrein panel [22] and the Prostate Health Index [23] – appear to add much needed specificity for the detection of high-grade (Gleason ≥7) prostate cancer at a diagnostic prostate biopsy, thereby potentially reducing the number of unnecessary biopsies performed. Advanced imaging modalities, including multi-parametric MRI, may also better refine the candidacy and yield of biopsy [24]. Among newly diagnosed patients, tumor-based risk stratification methods [25] and favorable long-term experiences with active surveillance [10] are poised to improve the confidence and quality with which incidental tumors are managed. Such measures, being currently clinically implemented, are highly promising means to cultivate better screening; these methods will highlight prostate cancer requiring attention while disregarding or proposing active surveillance of those that do not.

**F Schröder:** In my view, the time for population-based screening has not come and may never do so. The main reason for my pessimistic view on this issue is the high probability (of approximately 40 %) of diagnosing cancers which will not progress clinically, cause symptoms, or lead to death (overdiagnosis) [5]. While there are methods available to decrease overdiagnosis, such as the use of risk calculators and multiparametric MRI of the prostate, these have not been sufficiently validated in multicenter use to establish their accuracy in routine clinical practice. As a main contributor and former principal investigator of the ERSPC screening trial, I am delighted to see increasing worldwide acceptance of the recommendation of our group, including in US, European, and Russian guidelines to apply ‘shared decision-making’. We recommend the use of a procedure developed on the basis of ERSPC data, which is freely available on the website of the SIU [26].

**A Vickers and S Carlsson [to M Leapman and P Carroll]: Could you please clarify what you think current policy should be and whether all men should be screened?**

**M Leapman & P Carroll:** On the basis of randomized screening trials, PSA screening should be offered to healthy individuals without known risk factors for prostate cancer beginning at age 45 [5, 27]. No stark policy, however, will account for the complexities involved with screening and early diagnosis of prostate cancer. As a result, screening of asymptomatic men should be approached in the setting of a shared decision between a patient and physician cognizant of the individual’s age, health status, personal preferences, and risk factors including family history, race, prior PSA, and biopsy status. As noted above, the efficiency of screening can be improved and contemporary screening guidelines are incorporating refinements as suggested [28].

**A Vickers and S Carlsson: You say that “*discontinuing screening for those with low baseline risk profiles*[would]*significantly improve the efficiency of*[PSA]*screening*”. Could you give a specific example of the profile of a man for whom screening should be discontinued?**

**M Leapman & P Carroll:** Obviously, those in poor health or of advanced age do not benefit from early detection efforts. The optimal frequency of PSA testing has not been explicitly compared in a randomized fashion, but screening at 2–4 year intervals appears appropriate in low-risk patients based on PSA levels. Baseline PSA status may offer a valuable insight into a patient’s further risk for harboring or developing significant prostate cancer. Persuasive evidence from a Swedish population-based cohort examining PSA levels at age 60 suggests that men with levels <1 ng/mL possess a low (near zero) risk of prostate cancer death in extended follow-up [14, 17]. It would be reasonable to forego further screening of a 60-year-old without other known risk factors with baseline PSA <1 in the absence of cause.

**A Vickers and S Carlsson: You mention various methods to improve the risk benefit ratio of PSA screening, including restricting screening in older men, greater use of active surveillance, and discontinuing screening for some men at low risk. Do you think it will actually be possible to shift clinical practice in this way? For instance, radiation oncologists have a huge financial incentive to treat rather than send a patient back to the urologist for active surveillance. Do you think we can get radiation oncologists to use active surveillance routinely in low-risk patients? How can we actually get primary care physicians to screen the right men and avoid screening the wrong men?**

**M Leapman & P Carroll:** It is true that specialists of all types may have financial incentives to treat, a relationship that has been used to publically challenge practice patterns [29]. A more optimistic view of the primacy of sound clinical practice may be offered by encouraging trends in the state of Michigan, where nearly half of all patients diagnosed with low-risk disease are initially managed with active surveillance [30]. However, who should be entrusted with the stewardship of screening and early detection on a population level? Primary care physicians far outnumber urologists, yet may lack expertise in the subtleties of the disease. Clear and concise policy statements as well as better engagement and education of primary care physicians are essential in the faithful implementation of such a public health strategy, and should be coupled with close participation of specialists when warranted.

**A Vickers and S Carlsson [to F Schröder]: You state that the data do not support “*population-based screening*”. The meaning of this term is not entirely clear. It could be interpreted as “*every man getting a PSA test irrespective of preference*”, in which case, few would disagree with you: is there anyone who is proposing mandatory PSA testing?**

**F Schröder:** ‘Population-based screening’ has been introduced for several cancers in several countries. It always includes an offer to a risk group (e.g. men, persons of certain ages) and indicates an ‘offer’ that allows the person to be potentially screened to decide. No one proposes ‘mandatory screening’.

**A Vickers and S Carlsson**: **Your recommendation appears to be for shared decision-making. But what is the exact policy recommendation here? Is it that all men should be approached (e.g. at 50 or 55) and asked if they want to consider PSA testing? Or should this discussion only take place if initiated by the patient?**

**F Schröder:** The application of screening tests will always occur through patient initiative. Healthcare policy makers have, in my view, the duty to draw attention to testing which provides proven health advantages. The candidate can then decide by establishing a personal view matching the potential advantages and harms.

**A Vickers and S Carlsson:You recommend the use of a specific decision aid published by the SIU. Is your vision that this should be given to all men who are of PSA-screening age?**

**F Schröder:** High quality decision aid instruments should be freely available and easily accessible in many languages, but not given without request. Such a procedure would exert some unwanted pressure on persons at risk. The SIU decision aid [26] is only an example.

**A Vickers and S Carlsson:The section of your decision aid aimed at patients is non-quantitative, listing various advantages and disadvantages (e.g. cure a treatable cancer vs. unnecessary treatment of indolent disease). However, any course of action has pros and cons. If I choose to go for a walk right now, the advantage is that I get some fresh air and exercise and the disadvantage it that I might get shot. I can't make a decision about whether to go for a walk unless I know my risk of being killed (compare living in a peaceful area versus a war zone). More specifically, imagine it were the case that a terrific new marker was discovered as a reflex test that had 99 % specificity and sensitivity for lethal disease. None of the wording on the decision aid would change (there is still a risk of unnecessary treatment and a risk of false reassurance). How is a patient meant to weigh these pros and cons without some idea of risk?**

**F Schröder:** Weighing pros and cons of a medical procedure will always remain the responsibility of the person at risk. We, as doctors and urologists, have to help by addressing the questions arising after reading the first section of the decision aid, which is only one page. The two other sections are meant for the health professional. My personal view is that the pros and cons which are relevant to potential patients should be made widely accessible using all currently available evidence and instruments.

**A Vickers and S Carlsson**: **The decision aid is 20 pages long. Is it realistic to expect primary care physicians to read and remember so much information (e.g. rate of discomfort during biopsy of 55 %)?**

**F Schröder:** The section meant for the primary care physician is held very short for the reasons given. The urologist has to have a deeper insight in preparation of shared decision-making with potential patients.

## Who and when to screen, and not to screen, for prostate cancer: the skeptics’ view

We asked these authors, generally perceived as skeptical about prostate cancer screening, what they think current policy for prostate cancer screening should be. We then asked some follow-up questions.

**Fig. 5:**
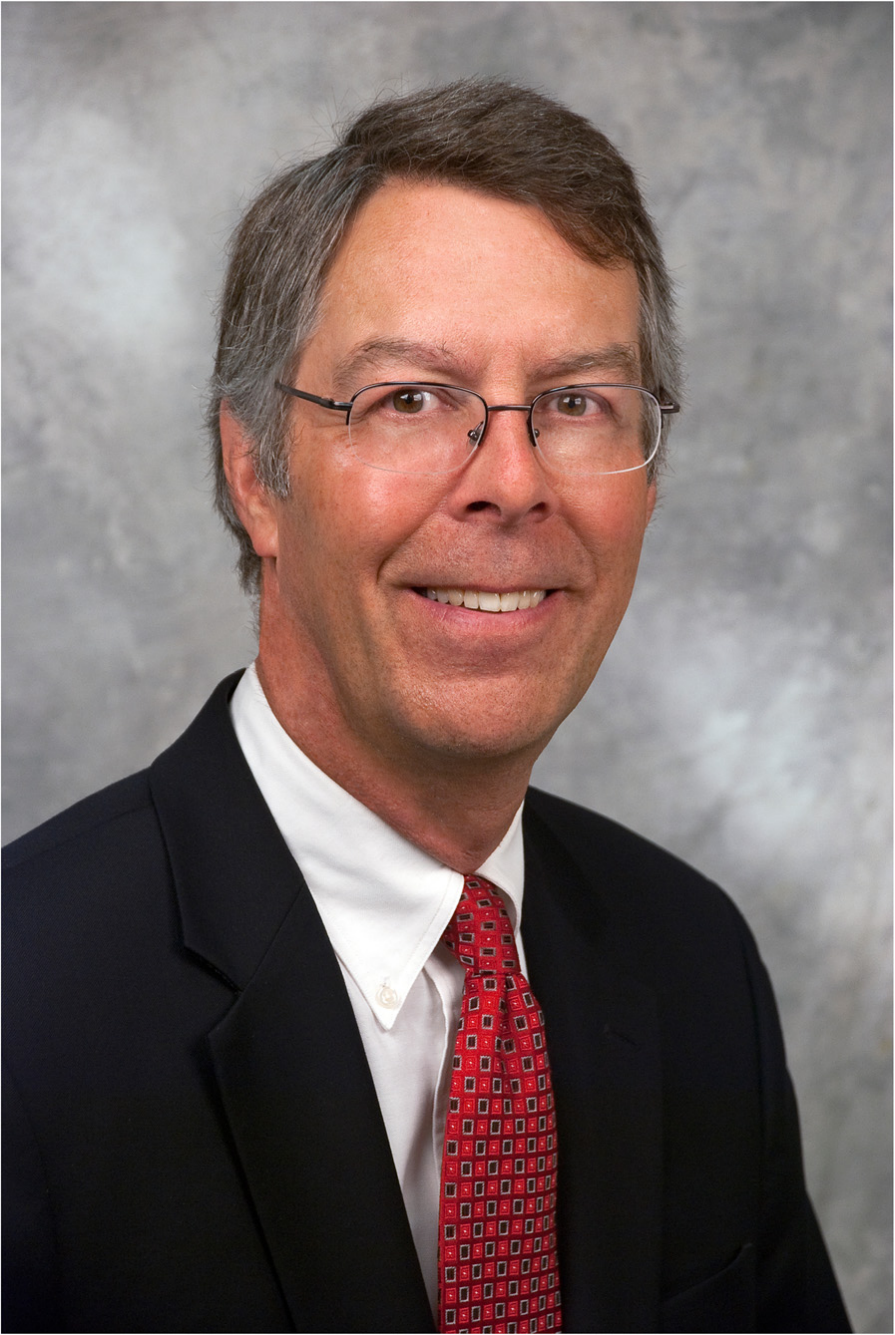
Peter C Albertsen is chief of urology and residency program director at the University of Connecticut Health Center in Farmington, CT. His research interests have focused on understanding the natural history of prostate cancer and the impact of treatment on health-related quality of life. He has published widely in the field of outcomes research in prostate cancer, including the impact of screening for prostate cancer. He is a past trustee of the American Board of Urology and has served as President of several national urological societies

**Fig. 6:**
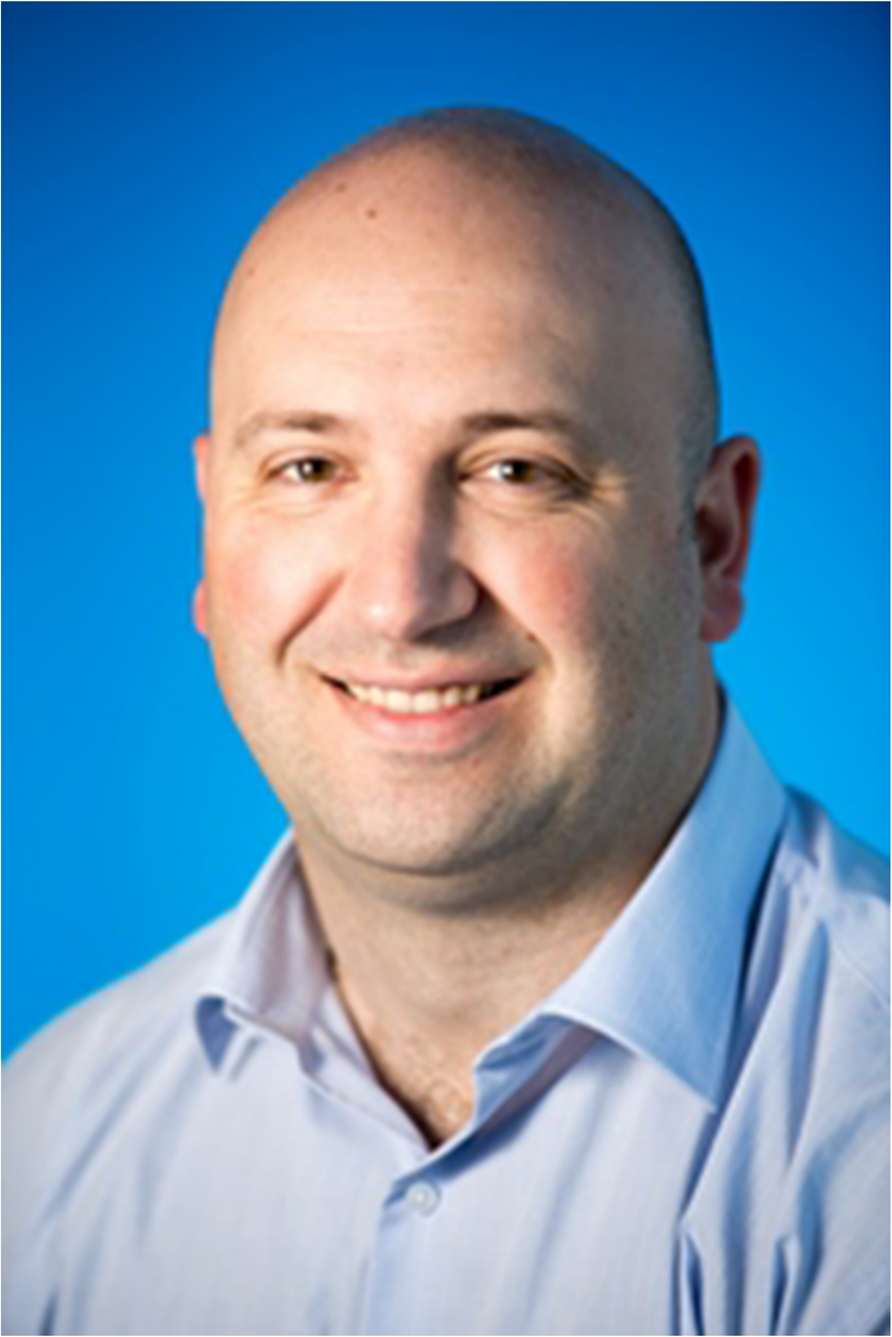
Dragan Ilic is Associate Professor of Evidence Based Clinical Practice at the Department of Epidemiology and Preventive Medicine, School of Public Health and Preventive Medicine, Monash University, Australia. He is a member of the Cochrane Urology review group and co-author of the Cochrane systematic review ‘Screening for Prostate Cancer’

**Peter C Albertsen (Fig. **5**) and Dragan Ilic (Fig. **6)

**A Vickers and S Carlsson: You have generally been perceived as an individual who is skeptical about prostate cancer screening. What do you think current policy should be; should there be no screening ever? That is, should PSA be avoided entirely in asymptomatic men, or should screening be restricted to certain subgroups and, if so, whom? Justify your answer with reference to the literature.**

**P Albertsen:** Few cancers generate as much controversy as prostate cancer concerning screening, diagnosis, and treatment. From 1977 to 2005, the lifetime risk of prostate cancer diagnosis in the US increased from 7.3 % to 17 % [31, 32]. During this same period, the lifetime risk of dying from prostate cancer fell from 3.0 % to 2.4 %.

My views on prostate cancer screening and treatment have been shaped by my training in urology at Johns Hopkins and epidemiology and public health at the University of Wisconsin, as well as by my patients. One patient in particular had a powerful influence. He was referred for treatment of a localized prostate cancer and underwent successful surgery. His postoperative PSA was undetectable and all surgical margins were negative. I told him he was cured only to have him return 2 years later with widespread metastases. I treated him successfully with anti-androgen therapy for an additional 16 years. According to the Halsted model of cancer progression he should have been cured by surgery [33]. I had the original specimen re-cut to document negative margins and concluded that we had a poor understanding of the natural history of this disease and the efficacy of treatment.

To address this problem, I developed a Markov model of prostate cancer progression. With the assistance of Jack Wennberg’s research group, the model was published in *JAMA* [34]*.* At the time, it was severely criticized by the urologic community, but when reviewed today the model bears a remarkable resemblance to data recently published by the SPCG-4 [35]. The sensitivity analysis indicated that data concerning the natural history of this disease was most critical to estimating the relative value of intervention. This is why I gathered data on the natural history of this disease from patients enrolled in the Connecticut tumor registry and published them in *JAMA* in 1998 and 2005 [36, 37].

My training in epidemiology taught me to view healthcare delivery from a public health perspective. Screening programs were best assessed by addressing four key questions: (1) Is prostate cancer a suitable disease for screening? (2) Is treatment for prostate cancer effective? (3) Is PSA an effective screening test for this disease? (4) Does screening result in any harm?

Early detection and treatment for prostate cancer is not a new idea. As early as 1905, Hugh Hampton Young suggested that a careful digital rectal examination could identify changes in prostate gland texture that could lead to the early diagnosis of cancer and appropriate intervention [38]. Only recently have we begun to appreciate that a large number of men harbor indolent disease, as clearly demonstrated by data from the Finasteride Chemoprevention trial [39]. Many pathologists now question whether Gleason 3 + 3 tumors are sufficiently aggressive to cause morbidity [40]. Indeed, recent data from the ERSPC trials suggest that half of all screen-detected cancers are indolent [5].

The efficacy of treatment also poses problems. Most urologists and radiation therapists assume that surgery and radiation are curative. However, what do the data say? The SPCG-4 suggests that some men are cured by surgery, but many men are not [35]. Further, men with high-grade disease often die from prostate cancer despite surgery and that surgery is primarily palliative for men aged over 65. With regards to radiation, even less information concerning its efficacy is available to date.

How well does PSA perform as a screening tool? Unfortunately, many lethal tumors produce low amounts of PSA and are missed by screening studies. Additionally, prostate enlargement, prostatitis, and surgical manipulation can lead to a significant number of false positive values. Finally, screening can result in considerable morbidity as documented by the USPSTF report [1].

Do I believe that PSA testing should be abandoned? Of course not, screening clearly benefits some men. This is why I helped draft and support the prostate cancer screening guidelines approved by the AUA [6]. Unfortunately, most clinicians focus primarily on the potential benefits of screening in individual patients and downplay the harms associated with testing as measured from a public health perspective. Before we can advocate for population-based screening, we must understand the natural history of the disease identified by testing and gain a better appreciation of the efficacy of treatment. This is why I have been an advocate of active surveillance for men with low-grade disease [9]. Data from the ProTECT trial should provide important new data concerning efficacy when published in the spring of 2016 [41]. Ideally, screening should only identify men destined to suffer from clinically significant disease and patients should only be offered treatments that yield substantial benefit. We still have a long way to travel to reach this goal.

**D Ilic:** Prostate cancer is a leading cancer affecting men worldwide [42]. Despite its high prevalence, the current evidence suggests that screening asymptomatic men for prostate cancer is not warranted [1, 43]. The most recent Cochrane systematic review identified five randomized controlled trials examining the effectiveness of screening [43]. A meta-analysis of data from those five trials determined no significant difference in prostate cancer mortality between men randomised to screening in comparison to those who were not (risk ratio (RR) = 1.00; 95 % CI, 0.86–1.17) [43].

Only two of the five randomized controlled trials included in the 2013 systematic review were determined to methodologically present a low-risk of bias [43]. The point of interest lies in the differing results and conclusions offered by two studies: the ERSPC and the Prostate, Lung, Colorectal and Ovarian (PLCO) cancer screening trial [27, 44]. The ERSPC study was the only study, out of the five included in the meta-analysis, to demonstrate a reduction in prostate cancer mortality from screening (RR = 0.84; 95 % CI, 0.73–0.95) [27]. Conversely, the PLCO study reported no benefit in screening (RR = 1.15; 95 % CI, 0.86–1.54) [44]. Criticisms of both the ERSPC and PLCO studies exist. The PLCO study has been criticized for the large number of participants entering the trial with a history of screening and the level of contamination by control participants continuing to be screened [43, 45, 46]. The ERSPC study has been critiqued for the variation in study protocols and their implementation across study sites [43, 46]. In 2014, the ERSPC published 13-year follow-up data, reporting a 21 % reduction in the risk of prostate cancer mortality through screening (RR = 0.79; 95 % CI, 0.69–0.91) [5]. A sub-group analysis of prostate cancer mortality by age at randomization identified a significant decrease in prostate cancer mortality in the 65–69 year age group (RR = 0.69; 95 % CI, 0.55–0.87). No statistically significant difference in prostate cancer mortality was observed between screening and control groups in men aged <54, 55–59, and 60–64 years [5]. The results also suggest that screening is not beneficial in men aged over 70 years (RR = 1.17; 95 % CI, 0.82–1.66). The ERSPC study authors concluded that, “*…the time for population-based screening has not yet arrived…*” [5].

Given that the current evidence does not support population-based prostate cancer screening, the question then turns to screening on an individual basis. For individual patients to make an informed decision, they must be aware of the benefits and harms associated with the diagnostic tests used when screening for prostate cancer. The PSA test is used as the common frontline test for prostate cancer; nevertheless, its sensitivity and specificity, as reported in the literature [47], varies widely. The potential for high false positive and significant false negative rates with PSA testing can lead to substantial harms, including overdiagnosis and overtreatment [1, 43, 47, 48]. Identifying a cancer that would never have become apparent in the absence of screening and then subjecting the patient to invasive treatment can lead to significant physical, emotional, and psychosocial harms [1, 43].

Age, family history, and ethnicity have been determined as risk factors for prostate cancer [49]. Whilst there is no evidence to support population-based screening, patients and clinicians should discuss the potential benefits and harms of screening as it relates to the individual patient [1, 43]. The use of decision aids and risk calculators can increase patients’ knowledge and decision-making to ensure that patients and clinicians engage in an informed, evidence-based decision grounded on the patient’s needs and circumstances [50].

**A Vickers and S Carlsson [to P Albertsen]: You argue that, “*before we recommend ‘population-based screening’ we must understand the natural history of the disease identified by testing and gain a better appreciation of the efficacy of treatment.*” Can you be a little bit more specific?**

**What are the precise gaps in our understanding of the natural history of prostate cancer that prevent us from developing optimal screening programs?**

**What research, exactly, must be performed to address these gaps? What are the specific gaps and associated research studies that need to be carried out as regards treatment efficacy?**

**P Albertsen:** For a screening program to be successful from a public health perspective (i.e. population-based screening) three critical issues must be satisfied: (1) the disease in question must pose a significant health burden, (2) the screening tool must be able to identify the disease sufficiently early in its natural history so that outcomes can be altered, and (3) a treatment must exist that can alter the outcome of the disease identified by screening. Many urologists believe that PSA testing satisfies these criteria; unfortunately, it does not – neither when it was proposed in the late 1980s, nor now.

PSA screening does increase the likelihood of finding a condition termed prostate cancer, but it does not discriminate between a disease representing a significant health burden and one that is essentially indolent. As noted in the most recent report by the ERSPC, approximately half of the cancers identified by PSA testing are Gleason 6 and are not destined to pose any significant clinical burden [5]. We also know that PSA testing among older men is unlikely to identify clinically significant disease in many men, yet most screening appears to be done in this age group [51]. The Finasteride Chemoprevention trial has provided critical information on the widespread prevalence of low-grade prostate cancer and the PIVOT trial and the SPCG-4 trial have provided much needed information on the natural history of prostate cancer [35, 52, 53] The ProTECT trial should add to this critical data when reports are published next year. While many of us recommend active surveillance for men with Gleason 6 disease, most of these men are still receiving aggressive interventions that often do more harm than good.

Finally, the third criterion is equally important. Most urologists assume that a radical prostatectomy is curative; nevertheless, data to support this assertion are missing. Currently, the best data have arisen from the SPCG-4 trial, which demonstrates that most men with high-grade disease do not benefit from surgery [35]. Prostate cancer mortality is similar for men undergoing surgery and those undergoing delayed androgen suppression. Surgery does appear to benefit men with intermediate-grade disease, but this is seen primarily among younger men rather than those aged over 65. It could be that the study investigators did not identify men sufficiently early during the course of their disease; however, the PIVOT trial has also raised questions regarding the efficacy of surgery [53], thus highlighting the importance of the ProTECT trial. Unfortunately, we have even less information concerning the efficacy of radiation in any of its forms [41].

What are the gaps in our understanding of the natural history of prostate cancer? We need a tool that can clearly indicate whether a man has a disease that will cause significant morbidity and death during his natural lifetime. We also need randomized trials that demonstrate how well our primary treatments, namely surgery and radiation, work to alter the natural course of prostate cancer. There is no substitute for this information.

**A Vickers and S Carlsson: One of the problems with waiting for new data is that it is rarely as definitive as anticipated. You say that the “*ProTECT trial should provide important new data*” in 2016, but that will be pretty early data – only 10 years of follow-up – and does not reflect best practice: a single PSA when a man is in his 50s or 60s, surgery from low-volume surgeons, and relatively low-dose radiotherapy. As regards the treatment trial in ProTECT, about three quarters of patients have low-grade disease, which we now believe should not be treated at all. Do you really think that ProTECT will provide enough data about screening and treatment to move forward with practice recommendations?**

**P Albertsen:** We currently have practice recommendations from the AUA that focus PSA testing on the group of men most likely to benefit. While I agree that research trials are rarely as definitive as anticipated, each new study helps refine our understanding of the efficacy of screening and treatment. The ProTECT study will publish 10-year data next year. I agree that this is a relatively short follow-up, but it is 10 years ahead of any randomized trial being conducted in the US. You state that the trial does not reflect best practice, but how can you be sure that PSA testing, as currently practiced in the US, is best practice (see response to question #1). You dismiss the trial because it is being conducted by ‘low-volume surgeons’. What evidence do you have that surgery is less efficacious among low-volume compared to high-volume surgeons? These surgeons know how to operate and have undergone quality assurance comparisons as part of the trial. Is that also true for high-volume surgeons in the US? Imagine that the high-volume surgeons had participated in a similar trial when PSA testing became widely adopted in the US. If this had been the case, we would now have a completed trial with a large sample size and 20-year follow-up. The UK government would rather invest in clinical trials to determine if screening and treatment works, than simply pay for surgery and radiation that urologists/radiation therapists claim has a major impact. Finally, I would be surprised to learn that three-quarters of the men enrolled in the trial have low-volume, low-grade disease. These men are usually identified following repeated PSA testing. Let’s review the results together in 2016. Without the European trials, including SPCG-4, ERSPC, and ProTECT, we would only be left with the PIVOT and PLCO data [54]. How would urologists debate prostate cancer screening and treatment with these data? You seem to advocate crafting screening and treatment policies with the hope that the tools and treatments work. I am a bit more skeptical given the history of breast cancer screening and management and that of other medical theories that have been discarded.

**A Vickers and S Carlsson: You say that “*many lethal tumors … are missed by screening*”. We all know that does sometimes occur, but do you have any good estimates as to how common it is? The Malmö studies suggest that the incidence of lethal disease in men with low PSA is actually extremely low.**

**P Albertsen:** I agree with your statement that the incidence of lethal disease in men with low PSA is extremely low. I also believe that the incidence of high-grade, lethal prostate cancer, is also very low in general. This latter statement carries significant implications on the performance of a screening test. When the incidence of the condition you are searching for is low and the pool of potential patients is very large, test performance becomes critical – false positives rapidly overwhelm true positives. You can reassure most men in their early 50s and even 60s that they will not die from prostate cancer in the absence of PSA testing and be right 97 out of 100 times because the lifetime risk of dying from prostate cancer is approximately 3 %. Unfortunately, we have all seen the young men in their 50s who present with Gleason 8–10 tumors and have extra prostatic extension by the time they undergo surgery. As a clinician, I wish we could find these tumors earlier and cure these men. As someone trained in epidemiology, I know that the ability to achieve this dream is very difficult, especially when the tests have modest sensitivity and specificity and the treatments are moderately effective (see responses to Questions 1 and 2) [1].

**A Vickers and S Carlsson: The original question asked “*Should PSA be avoided entirely in asymptomatic men, or should screening be restricted to certain subgroups and, if so, whom?*”. You seem to agree with the latter: you say that screening should not be abandoned because it “*clearly benefits some men*”. So which men should be screened?**

**P Albertsen:** Based upon what we know in 2015, PSA testing does benefit some men. For this reason I strongly support the recommendations of the AUA. These guidelines were based extensively on the data provided by the ERSPC. However, we need to do better. I am aware of your work with Hans Lilja, suggesting that a PSA value at age 50 is predictive of the long-term probability of developing clinically significant prostate cancer [18]. I believe we can refine the group of men who should be tested. We also need to incorporate the natural increase in PSA that comes with prostate enlargement that occurs when a man ages through his 50s and 60s. A graphic chart that tracks PSA levels or, possibly, percent free-PSA or the new prostate health index test against age and prostate volume, similar to a pediatric growth chart, might be helpful. Men consistently falling outside the 90th percentile, for example, might undergo MRI testing before considering a prostate biopsy. All of these refinements should be aimed at lowering the incidence of low-volume, low-grade cancer. In my mind, we have yet to define a best practice screening and treatment algorithm. While the AUA recommendations are a start, we have not agreed upon the test(s) needed, the frequency of their application, the value of imaging versus biopsies, nor on which treatments work best for which patients. These are the research gaps that should be addressed by trials. Unfortunately, these trials are often difficult to conduct in the US because of perverse economic incentives and clinicians who are convinced that current practices are the preferred standard of care.

**A Vickers and S Carlsson [to D Ilic]:You seem to base your conclusions on the Cochrane meta-analysis. However, as you mention, three of the studies were associated with a high-risk of bias. Moreover, one of these studies did not even include PSA screening until very late in the trial. Do you think it is valid to report a risk ratio from a combined analysis including poor quality studies?**

**D Ilic:** The meta-analysis of all five studies provides a broad overview of the evidence available on the topic. The Cochrane review cited provides a sensitivity analysis in which these poor quality studies are removed and their impact upon the overall meta-analysis can be identified. It is interesting to note that, even following their removal, the sensitivity analysis with the two ‘good’ quality trials was not greatly affected (RR = 0.96; 95 % CI, 0.70–1.30 (‘good quality trials’) vs. RR = 1.00; 95 % CI, 0.86–1.17 (‘all trials’)) [43].

**A Vickers and S Carlsson: You imply that the key consideration is to compare the results of the PLCO and ERSPC studies. These studies addressed very different study questions. The ERSPC was a trial of screening versus no screening; the PLCO was explicitly described by the investigators as a trial of ‘systematic’ vs. ‘opportunistic’ screening. Would we expect these studies to have results that are in any way comparable? Do you think it makes sense to combine the results meta-analytically? By way of analogy, if we had a trial of aspirin vs. no aspirin for pain, and another trial of systematic aspirin vs. aspirin as needed, do you think we could combine the trials to get an estimate for aspirin effectiveness?**

**D Ilic:** The aim of performing a meta-analysis with these two studies is to increase the power, improve precision, and hopefully have the evidence to answer such a controversial question. In performing a meta-analysis, careful consideration is given to the impact of clinical, methodological, and statistical heterogeneity upon the outcome [55]. The PLCO study has been described as outlined above, partly due to the high rate of contamination reported and the potential for such contamination to mask any clinical benefit from screening. Methodological heterogeneity has been reported for the ERSPC study, with significant variation amongst study sites with respect to screening protocol, contamination, and follow-up [5]. In performing a meta-analysis of these studies, the assumption is that their objectives, study design, participants, interventions, outcomes measured, and follow-up are sufficiently homogeneous to allow the pooling of data.

**A Vickers and S Carlsson: You say: “*No statistically significant difference in prostate cancer mortality was observed between screening and control groups in men aged less than 54, 55–59, and 60–64 years*”. What conclusion do you draw from that?**

**D Ilic:** The rate ratios (RR) for the respective age groups are as follows, <54 (RR = 0.84; 95 % CI, 0.28–2.49); 55–59 (RR = 0.81; 95 % CI, 0.93–1.03); and 60–64 (RR = 0.90, 95 % CI, 0.71–1.15). These results would indicate that there is no evidence to support the hypothesis that screening significantly reduces the risk of prostate cancer mortality in these groups. For example, the risk ratio and confidence intervals for the <54 age group indicate great variability for the potential of benefit and harm as a result of screening. It should be noted that the AUA recently modified their guideline recommending against PSA screening in men under 40 years of age, and in men aged 40–54 years at average risk. For men aged 55–69 years, a shared approach to decision-making was advocated [56].

**A Vickers and S Carlsson: You state that “*patients and clinicians should discuss the potential benefits and harms of screening as it relates to the individual patient*” and then cite two papers: your own review showing no benefit to screening and the USPSTF report that explicitly stated that there was a reasonable degree of ‘certainty’ that screening does more harm than good. So what should clinicians tell patients about the harms and benefits of screening?**

**D Ilic:** Clinicians should refer to the ERSPC and PLCO studies, and their various strengths and limitations. The potential benefit of screening (as reported by the ERSPC) is a reduction in prostate cancer-specific mortality (0.11 per 1,000 person-years, or one prostate cancer death averted for every 781 men invited for screening) [5]. The PLCO study (and others) would suggest no reduction in prostate cancer mortality from screening. Potential harms include overdiagnosis, implications of false-positive test results, adverse events from further testing including hematospermia, hematuria, infection, bleeding, urinary difficulties and anxiety [1, 43, 56]. Such detailed information can be difficult to convey to patients in a short period of time during a consultation – hence the need for greater use of decision aids and other patient education strategies to ensure that patients are truly informed of the benefits and risks of screening.

**A Vickers and S Carlsson: You cite a review on the operating characteristics of PSA to suggest that its sensitivity is questionable. This review used the presence of prostate cancer as an endpoint. Many would argue that it is rather irrelevant whether PSA has good or bad sensitivity for prostate cancer, because most prostate cancer is indolent. The key question is whether PSA is sensitive for potentially lethal disease. What is your view on whether PSA is a good predictor for aggressive prostate cancer?**

**D Ilic:** The PSA test is currently the frontline test that clinicians rely on with respect to diagnosing prostate cancer. However, the PSA test is problematic in distinguishing whether the cancer will become lethal in men diagnosed with localized prostate cancer. The PSA test has been demonstrated to be a poor predictor for detecting lethal prostate cancer, as evidenced by men who have been diagnosed with localized prostate cancer and managed by watchful waiting [57]. Modifying existing strategies for using the PSA test, such as interval times and thresholds for biopsy, have been proposed, although greater emphasis is currently placed on the development of new markers that could differentiate between indolent and potential lethal cancers [49].

## Who and when to screen, or not to screen, for prostate cancer: the decision-analytical view. Don’t throw the baby out with the bathwater

We asked these authors, involved in implementing shared decision-making in primary care, what they think current policy for prostate cancer screening should be. We then asked some follow-up questions.

**Fig. 7:**
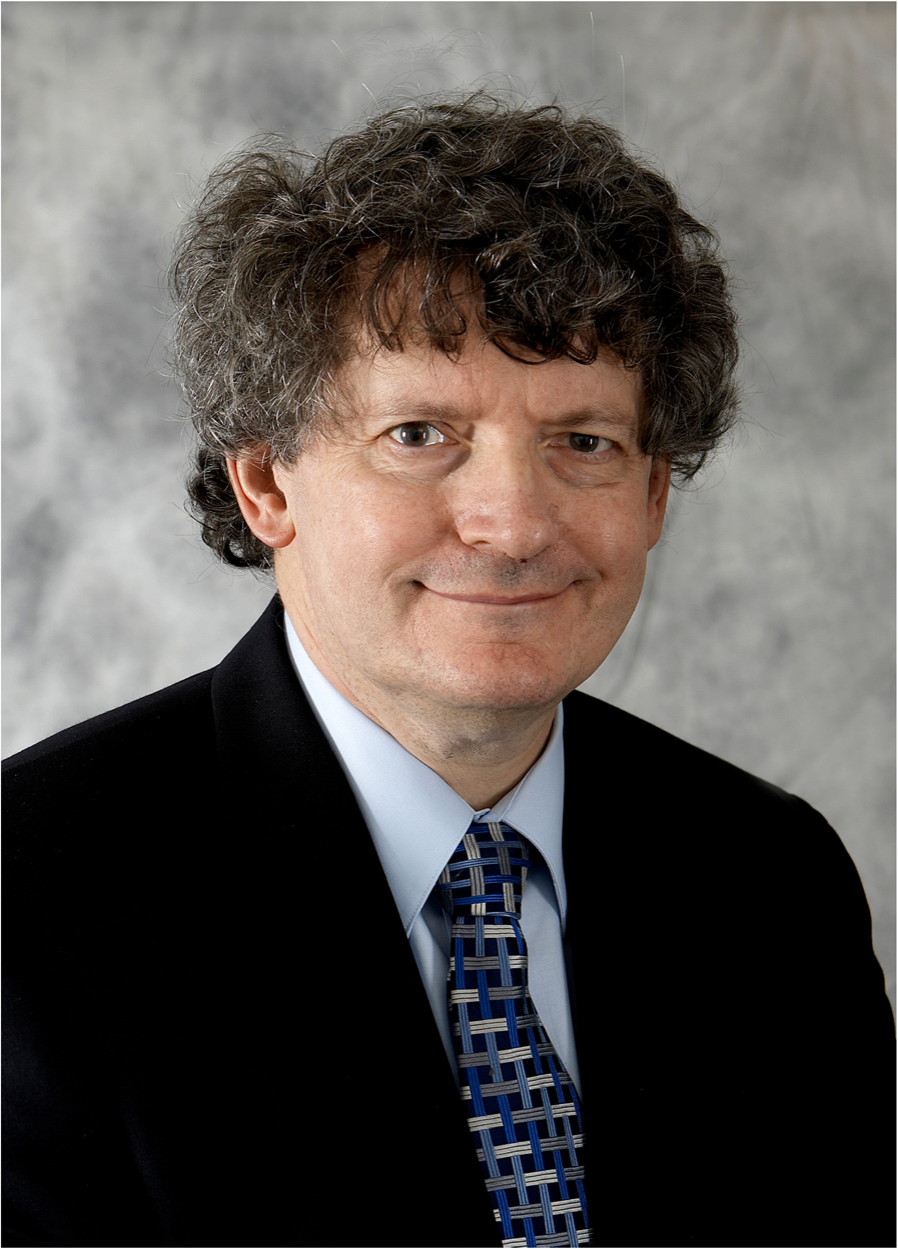
Michael Barry is president of the nonprofit Informed Medical Decisions Foundation and Chief Science Officer at Healthwise. Healthwise’s mission is to help people make better health decisions. He is a past president of the Society for Medical Decision Making and the Society of General Internal Medicine in the US. He has led many prominent research studies including the Patient Outcome Research Team for Prostatic Diseases. He continues to practice primary care and serves as medical director of the John D. Stoeckle Center for Primary Care Innovation at Massachusetts General Hospital. He is also a part time professor of medicine at Harvard Medical School and a Master of the American College of Physicians

**Fig. 8:**
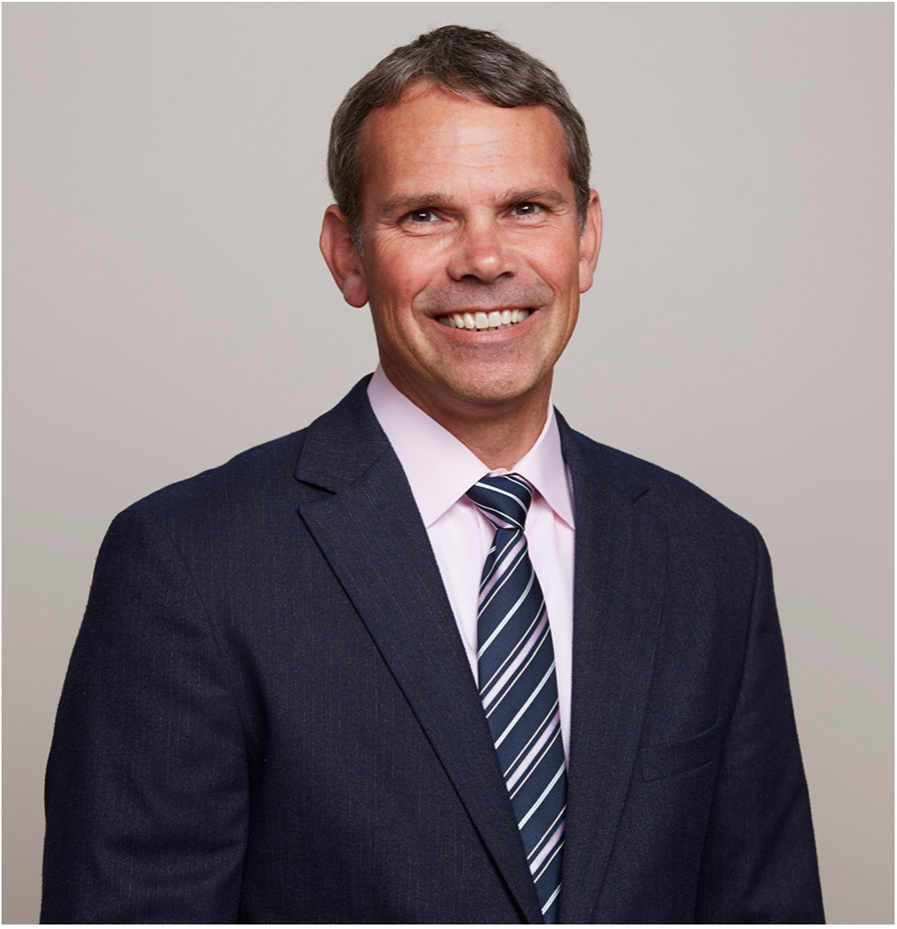
Dominick L Frosch, PhD, is a fellow in the Gordon and Betty Moore Foundation’s Patient Care Program and associate professor of medicine at the University of California, Los Angeles. He has conducted several randomized controlled efficacy and effectiveness trials of patient decision support for PSA screening and has led efforts to implement patient decision support in primary and specialty care. He currently serves as deputy editor for the *Journal of General Internal Medicine*

**Michael Barry (Fig.** 7**) and Dominick Frosch (Fig. **8)

**A Vickers and S Carlsson: You are involved in implementing shared decision-making in primary care. What do you think current policy regarding PSA screening should be? Do you think that all men should be screened, should there be no screening at all, or should PSA testing be restricted to certain groups? If so, whom? Justify your answer with reference to the literature.**

**M Barry and D Frosch:** In the early days of the development of clinical practices guidelines, Dr. David Eddy emphasized the importance of including the perspective of informed patients in the guideline development process. For example, a standard for or against an intervention would require that, *“…at least 95 %, perhaps even 99 %, of people who are candidates for the intervention should agree on the desirability* [or undesirability] *of its outcomes*” [58]. Current evidence from prostate cancer screening trials, in our minds, does not justify a standard to either ‘screen everyone’ or ‘screen no one’, at least for the pre-specified core group of men aged 55–69 for which a small but finite and statistically significant reduction in prostate cancer mortality was found in the ERSPC. In the latest follow-up from the ERSPC, at a 13-year mean follow-up, PSA screening reduced the risk of dying of prostate cancer from 0.61 % in the intervention group to 0.49 % in the control group for men aged 55–69, leading to a statistically significant 1.28 fewer prostate cancer deaths for every 1,000 men randomized. On the other hand, not surprisingly, screening increased the risk of being diagnosed with prostate cancer, from 6.1 % to 8.0 %. Moreover, the cumulative risk of a positive PSA test in the intervention group was about 17 %, with 82 % of these men receiving a biopsy [5]. This higher risk of prostate cancer diagnosis comes with considerable overtreatment with its attendant side effects, particularly of incontinence and sexual dysfunction, even more so in the US, where aggressive treatment of prostate cancers is routine. In the US PIVOT trial comparing radical prostatectomy versus observation of men randomized to surgery, 17 % had incontinence at 2 years versus 6 % with observation, and 81 % had erectile dysfunction compared to 44 % with observation [53].

Different men will see these tradeoffs between benefits and harms differently. Nine efficacy trials indicate that, when men are fully informed about the benefits and risks of prostate cancer screening using a decision aid, interest in the PSA test decreases [50]. However, even among men who can answer all questions correctly on a knowledge test about PSA screening, about a third still want a PSA test [59]. We believe both the clinical evidence and the equally important evidence about preference distributions among well-informed men supports a shared decision-making strategy about PSA screening for men aged 55–69, as recommended in the clinical practice guidelines by both the AUA and the American College of Physicians. Decision aids can be used to make this approach practical in the busy world of primary care. Shared decision-making is also important in making treatment decisions for men who choose screening and are diagnosed with prostate cancer; helping men understand that some form of observation, including active surveillance, is the optimal treatment for most PSA detected cancers can help break the cycle linking prostate cancer overdiagnosis with overtreatment [60]. Shared decision-making can help avoid throwing out the baby – and the PSA test – with the bathwater.

**A Vickers and S Carlsson: You make a strong case that the benefits of PSA testing, in terms of reduced mortality, do not obviously outweigh the harms, in terms of overdiagnosis and overtreatment. Specifically, you say that the harms are a particular problem because “*aggressive treatment of prostate cancers is routine*”. Your solution hinges on understanding the preferences for individual men. Why not focus on harm reduction instead? By way of analogy, the benefits of eggs for breakfast is that they are nutritious; the problem is that I could get egg on my tie. I could do a ‘shared decision-making’ where I consider how much I like eggs versus how much I care about my appearance. However, would it not be more effective just to eat more carefully or only put on my tie after breakfast? Why not put efforts into reducing overdiagnosis and overtreatment rather than treating these as a fact of life (‘routine’ in the US) and then applying preference-based approaches?**

**M Barry & D Frosch:** Although there is growing recognition in some quarters that prostate cancer is subject to substantial overdiagnosis and overtreatment, at present, it is difficult to conclude that there is consensus on this issue. Two recent reports paint a conflicting picture of the use of active surveillance in managing low-risk prostate cancer. One report, drawing on the US National Cancer Database, concluded that active surveillance remains substantially underused [61]. Another report, published one week later and drawing on the CaPSURE database, concluded that substantial progress has been made increasing the use of active surveillance [62]. The issue of patient acceptance of conservative approaches to managing cancer is further complicated by a long history of promoting cancer screening, with slogans such as ‘do not delay’ [63]. As a result, enthusiasm for cancer screening, and perhaps treating it aggressively, is higher than warranted among the general public and a recent study suggests that cancer screening is viewed as a moral obligation, rather than a deliberate decision that requires careful consideration of how the potential benefits and risks of screening align with individual patient preferences [64, 65]. Furthermore, the American public is acutely sensitive to any suggestion that access to medical treatment might be restricted or withheld. Thus, engaging patients in shared decision-making is perceived as a palatable approach to reducing overdiagnosis and overtreatment by sensitizing patients to important nuances of risks and benefits that should enter cancer screening and treatment decisions. Additional tests that could reliably distinguish aggressive from indolent cancers would be helpful, but these are not yet available. Implementing shared decision-making may be challenging, but changing clinician behavior is no less so. Arguably, attempts to address this problem should ideally focus on both sides of the equation.

**A Vickers and S Carlsson: You say that decision aids can make shared decision-making “*practical in the busy world of primary care*”*.*Prostate cancer decision aids have been around for a long time and they are not widely used. There is also evidence that, whatever their effect on decisional conflict and regret – two highly problematic endpoints – decision aids do not improve shared decision-making[**66**]. Do you really see a future where the millions of*US*men reaching screening age each year are routinely referred to decision aids before PSA screening?**

**M Barry & D Frosch:** It is important to recognize that providing decision aids to patients does not in itself constitute shared decision-making. Rather, these tools are intended to support a shared deliberative process by helping to inform a patient about the specific potential benefits and risks of PSA screening [67]. Achieving shared decision-making requires a physician who is appropriately trained and willing to engage patients in the necessary deliberative process. Making shared decision-making a routine process ultimately requires a significant cultural shift, both on the part of practicing clinicians and on that of patients, who, for the most part, have been socialized into a paternalistic medical culture. While this cultural transformation is arguably underway, considerable work remains, including systemic transformation of clinical processes and workflows to enable shared decision-making, as well as the development and implementation of corresponding quality measures [68]. The Cochrane review summarizing all randomized trials of patient decision aids indeed shows reduced proportions of people who are passive in decision making when decision aids are used (summary risk ratio, 0.66; 95 % CI, 0.53–0.81, n = 14 trials) [50]. A recent systematic review of surveys of patient preferences for participation confirms the growing public desire for shared decision-making. While the majority of respondents desired shared decision-making in 50 % of surveys conducted prior to 2000, for surveys conducted after 2000, this number increased to 71 % [69]. We can definitely envision a future when millions of patients engage in shared decision-making around cancer screening with their clinicians, facilitated by the availability of high-quality decision support. We believe that the translation of this vision into reality is underway, but considerable work remains and the path to systemic culture change in which fateful decisions are made in partnership between patient and clinician will not always be smooth or linear.

**A Vickers and S Carlsson: You cite evidence that use of decision aids reduces prostate cancer screening rates. This might well be seen as unsurprising given that these aids were implemented some years ago and long-term follow-up from ERSPC – perhaps the major reason why a man might consider PSA – has only just been made available. Indeed, some decision aids include directly false statements against PSA. For instance, the decision aid used in the study by Sheridan et al. [**66**] stated that there is no way of telling whether a cancer found by screening is dangerous or not. Do the lowered rates of screening following implementation of decision aids reflect an inherent property of decision aids or the content of the decision aids currently available for prostate cancer?**

**M Barry & D Frosch:** Content is important and decision aids need to be kept up-to-date with the latest evidence (see comment below regarding decision aid certification). Nevertheless, whether evolving evidence regarding the benefits and harms of PSA screening will change the proportion of men who decide for or against screening remains to be seen. The absolute risk reduction in prostate cancer mortality with longer follow-up of the ERSPC has increased from 0.71 per 1,000 men randomized at 9 years to 1.28 per 1,000 men randomized at 13 years [5, 70]. Whether such an increase in absolute difference will change many men’s minds, particularly when the risks are to be faced in the nearer term compared to the delayed benefits, remains unknown. Even at 13 years, the ratio of the number of men who must face a diagnosis of prostate cancer to the attendant risks of treatment remains high, at 27 for each prostate cancer death averted.

**A Vickers and S Carlsson: We have previously highlighted that current decision aids[**8**]used questionable estimates of risk and often disagree with one another (e.g. one decision aid gives a risk of overdiagnosis at approximately four-fold to another). Further, several decision aids are available. Which should a clinician use?**

**M Barry & D Frosch:** With the proliferation of decision aids from a wide range of developers, both commercial and academic, there is an urgent need for a certification process to ensure that decision aids present comprehensive and valid information [71]. The state of Washington, which passed legislation in 2007 offering a higher level of malpractice protection to physicians who engage patients with certified decision aids, is currently at the forefront of developing such a process [71, 72]. While this does not solve the immediate problem facing a clinician choosing a decision aid for patients today, it is the reflection of an evolving marketplace that is gradually maturing. The Affordable Care Act recognized the need for a certification process, but thus far the US Congress has not appropriated resources to support its development.

**A Vickers and S Carlsson: Quantitative decision aids require that patients integrate up to ten risk estimates for very different events (including pulmonary embolism, biopsy symptoms, erectile dysfunction, and prostate cancer death). Do you think this is feasible for most patients or only for a small proportion?**

**M Barry & D Frosch:** Over the past several decades, researchers have developed numerous innovative ways of communicating risk information to patients with a range of numeracy and health literacy skills. We believe these tools can enable us to communicate and integrate complex risk information to a wide range of patients [73]. Some of the risks cited above, such as the risk for pulmonary embolism, seem more appropriate for decision aids addressing prostate cancer treatment, rather than screening. Nonetheless, a rigorous certification process can help ensure not only that the information provided is accurate and valid, but that appropriate methods are used to present the information to maximize understanding by patients from a wide range of backgrounds, including those with lower levels of health literacy and numeracy.

## Discussion

**Responses to the Q & A**

**Sigrid Carlsson and Andrew Vickers**

In this Q & A, we asked seven experts who have studied PSA screening to provide their view on what prostate cancer screening policy should be. Our original aim was to assess whether a middle ground could be found, for example, by asking proponents where PSA testing should be restricted and skeptics where it might be of value. We believe we were successful in finding this middle ground. There was unanimity among our respondents that PSA should not be implemented routinely at the population level. In contrast to, for example, cervical cancer screening, we should not expect a situation where most individuals in the population at risk would receive a PSA test as a routine part of care. Instead, all experts agree that screening should take place only as shared decision-making between an individual man and a healthcare provider. There was also unanimity that prostate cancer screening was associated with important harms and that increased use of active surveillance for low-risk disease is required.

With the exception of Dr. Ilic, there was also agreement that prostate cancer screening does indeed reduce prostate cancer mortality. We find ourselves critical of Dr. Ilic’s response – we did not feel that his response was sufficiently clear regarding the critical issue of whether it is justified to combine the PLCO [44] and ERSPC results [5], referring only to the value of meta-analysis in general. In answering a question about the proven value of PSA for predicting long-term risk of lethal disease at the population level, Dr. Ilic rather focused on the value of PSA in men diagnosed with prostate cancer. Further, some differences in emphasis were observed; for instance, Dr. Albertsen is clearly more concerned about the current state of the data compared to ourselves or Drs. Leapman and Carroll. However, such differences generally represent a greater issue for research than for clinical recommendations.

The major area of disagreement between the respondents focused on how shared decision-making should be performed; in particular, whether formal decision aids should be given to patients and whether the key driver of decision making should be preference or behavior. Drs. Leapman and Carroll, as well as Dr. Albertsen and ourselves, focused on behavior rather than preference, believing that the net benefit of screening will be optimized by the increased use of active surveillance and limiting screening in older men. Conversely, Drs. Schröder, and Barry and Frosch, emphasized the use of preference-oriented decision aids, whereas Dr. Ilic focused on informed consent based on an in-depth explanation of current research findings. Dr. Schröder indicates that it is the patient himself who has to take the initiative and raise the topic of screening; if he does so, he should be given a short decision aid that describes, in general terms, the pertinent harms and benefits; it is then “*the responsibility of the person at risk*” to weigh those pros and cons. Drs. Barry and Frosch similarly appear to double down on formal decision aids when challenged. When questioned as to whether focus should be placed on changing clinician behavior, rather than assessing preference in the context of fixed clinician behavior, they stated that there is insufficient consensus that overdiagnosis and overtreatment is a problem in prostate cancer. They do not directly provide evidence to support this assertion, referring instead to the literature on public perceptions of screening. We have sympathy for the argument that lay ideas about cancer complicate efforts to combat overtreatment. However, we disagree that there is a lack of consensus on overtreatment or that clinician behavior is overly resistant to change. The authors cite two papers on changes in the use of active surveillance and conclude that results are conflicting. However, the paper suggesting little progress [61] only includes data up to 2011. The article including more recent data involved 45 predominately community-based urology practices in 28 states across all regions of the US and indicated that the use of active surveillance for low-risk disease increased from around 10 % in 2000–2009 to 40 % in 2010–2013 [62]. This number is still far too low and more work needs to be performed to change practice. We accept that such work is challenging and that it will meet resistance. However, we would argue that it will lead to a far greater health gain than a focus on preferences.

Drs. Barry and Frosch also appear optimistic when questions were raised about the value of decision aids. There is a large number of conflicting decision aids available: which should we choose and how complex do they need to be? Can patients really integrate a large number of risk estimates for different events and are these estimates evidence based? Drs. Barry and Frosch tell us, in general terms, about new initiatives to develop ‘certification’ processes for decision aids and suggest that “*innovative ways of communicating risk*” will allow patients to understand the complexities of screening. This may well be true, but, as they themselves acknowledge, that leaves us unable to advise our patients at the current time.

In a question to Drs. Barry and Frosch, who support preference-oriented decision aids, we used a breakfast analogy. Eating eggs is associated with benefit (taste, nutrition) and harms (risk of getting egg on your tie). This trade-off can be posed either in a preference framework (how does the individual man feel about the taste and nutritional value of eggs compared to having a smart appearance?) or a behavioral one (what can a man do to prevent getting egg on his tie?). Our own view is that, while preference does indeed play a role, its value is dwarfed by the importance of clinician behavior. Making choices congruent with preferences does affect quality of life, but its effect is small compared to, for example, an unnecessary radical prostatectomy or using PSA to detect a cancer in an older man highly unlikely to benefit from screening. Assessing preferences is also rather challenging: how will a man know how he will feel about being impotent or incontinent, or for that matter, metastatic prostate cancer, if he has never experienced it? Men who initially tell us “*I’d rather die than be impotent*” generally report a good overall quality of life 5 years after treatment, even with erectile dysfunction; a man who says “*I’ll go when god calls me*” may have little idea just how miserable it is to die of prostate cancer.

We also have considerable concerns with proposals, such as that made by Dr. Ilic, that doctors discuss the numerical results and methodologic limitations of randomized screening trials with patients. We note that many issues in the interpretation of these trials are extremely complex – contamination, pre- vs. post-randomization consent, and time-dependent effect sizes to name but three – with even experts struggling to grasp key points. Interpretation of trials also requires considerable numerical abilities: take for instance, a risk reduction of 0.11 per 1,000 men or a number needed to screen of 781. It is also unclear how a man should integrate multiple conflicting risks and benefits into one discrete choice: agree to or decline a PSA test.

It is also interesting that many decision aids present virtually all possible downstream consequences of a PSA test such as transient hematospermia from prostate biopsy or pulmonary embolism after surgery. We note that doing so is far outside typical medical practice. Is there any other routine screening test for which we have these types of complex discussions? Take the following imaginary conversation between a doctor and, for example, a 55-year-old man presenting for a general health check: “*Mr. Jones, before I draw your blood or put the cuff around your arm, I should warn you that in the event you have high cholesterol or high blood pressure, you may, in the future, need medication, such as a statin for high cholesterol or a diuretic, ACE-inhibitor, β-blocker, or calcium channel blocker for high blood pressure. All of these medications may give you side-effects. These include tiredness, hypotension, bradycardia, leg pain, vertigo,* [list continues]. *The probability of each of these side effects is as follows, for instance, for atenolol: tiredness (13 %), hypotension (10 %), bradycardia (8 %), depression (3 %)* [list continues]; *for a thiazide diuretic, there are 34 listed adverse effects* [74]. *On the other hand, medications reduce the risk of future problems with your heart and circulatory system. For instance, β-blockers are associated with a reduced risk of total cardiovascular disease, mainly stroke, in the order of seven fewer cardiovascular events per 1,000, compared to placebo* [75], *although effects on overall mortality are controversial. You should also know that the different drugs have not been fully compared to see which is most effective. So, Mr. Jones shall I go ahead and measure your cholesterol and blood pressure or not?”* Such a conversation is obviously absurd. We also note that few doctors, if any, mention even the possibility of urinary side effects (frequency from diuretics) or erectile dysfunction (from β-blockers) as a downstream consequence of a blood pressure measurement, let alone quantify the magnitude of these risks. Yet, exact quantification of urinary and sexual side effects is seen as pretty much mandatory for PSA decision aids.

## Conclusions

In summary, we agree with our expert authors that prostate cancer screening can save lives, that this comes at a high cost in terms of overtreatment and overdiagnosis, and that shared decision-making is necessary before administering a PSA test. Nevertheless, we disagree with Drs. Schröder, Barry, and Frosch in that PSA screening is predominately a matter of preference that should be addressed by handing out decision aids assessing preference. Further, we disagree with Dr. Ilic that it is appropriate to discuss the results of complex trials with patients considering a PSA test.

PSA screening is not a single defined intervention: it can be practiced in numerous different ways and there is good (if inevitably incomplete) research data to suggest which would be more helpful and which more harmful. It is time to end sterile, for-and-against debates and focus on making sure that contemporary PSA screening practice follows best evidence.
